# Long-term visual outcomes in spasmus nutans

**DOI:** 10.1186/s12886-024-03494-7

**Published:** 2024-06-12

**Authors:** Lauren Hennein, Gena Heidary, Eric D. Gaier, Ryan Gise

**Affiliations:** 1 Ophthalmology Division, Rady Children’s Hospital San Diego, San Diego, CA USA; 2 Viterbi Family Department of Ophthalmology, University of California San Diego, La Jolla, CA USA; 3 Department of Ophthalmology, Boston Children’s Hospital, Harvard Medical School, 300 Longwood Avenue, Boston, MA 02115 USA; 4 Picower Institute of Learning and Memory, Massachusetts Institute of Technology, Cambridge, MA USA

**Keywords:** Spasmus nutans, Amblyogenic refractive error, Strabismus, Amblyopia

## Abstract

**Background:**

The long-term visual outcomes in spasmus nutans patients is largely unknown. The purpose of this study was to characterize visual outcomes and identify comorbid ophthalmic conditions in patients with spasmus nutans.

**Methods:**

We retrospectively reviewed the charts of consecutive patients diagnosed with spasmus nutans between 2000 and 2020. Demographic information, ophthalmic characteristics, and neuroimaging results were assessed over time.

**Results:**

Of the 32 patients included in the study, 13 (41%) were female. Underlying medical conditions included a diagnosis of Trisomy 21 in 6 (19%) and prematurity in 8 (25%). Twenty-one patients (66%) self-reported as a race other than Caucasian. 18 patients (56%) had non-private health insurance and 1 (3%) was uninsured. Mean age at diagnosis and resolution were 16 months (range 45 months) and 48 months (range 114 months), respectively. All 32 patients had nystagmus, 31 (97%) had head nodding and 16 (50%) had ocular torticollis. Mean follow-up was 66 months (range 185 months). On initial presentation, 6/32 (19%) had an amblyogenic refractive error and mean best-corrected visual acuity (BCVA) in the better-seeing eye was 0.78 Logarithm of the Minimum Angle of Resolution (LogMAR) (range 1.24). In a sub-analysis that included patients with > 1 exam (*n* = 23), 17/20 (85%) had an amblyogenic refractive error and mean BCVA in the better-seeing eye was 0.48 LogMAR (range 1.70). At the final exam, 12 patients had measurable stereopsis, eight had strabismus, and three had undergone strabismus surgery. Eight patients required treatment for amblyopia.

**Conclusions:**

We found a high prevalence of amblyogenic refractive error, strabismus and amblyopia among patients with spasmus nutans. Children with spasmus nutans benefit from ongoing ophthalmic follow-up until they are past the amblyopic age range, even after resolution of nystagmus.

**Supplementary Information:**

The online version contains supplementary material available at 10.1186/s12886-024-03494-7.

## Background

Spasmus nutans is classically characterized as the clinical triad of nystagmus, head nodding, and an anomalous head position. The nystagmus of spasmus nutans is typically asymmetric, fine amplitude, and high frequency [1, 2]. The condition has been proposed to begin in infancy (before age 12 months) with anticipated resolution by two years of life [3, 4]. The pathogenesis of spasmus nutans is unknown [3, 4], . but lower socioeconomic status has recently been identified as a risk factor for spasmus nutans [5]. 

Patients with spasmus nutans have traditionally been counseled on its benign, self-limited nature. However, data on long-term visual outcomes in these patients is limited and inconsistent with respect to the impact of asymmetric nystagmus, contributions of associated strabismus, and refractive error [3, 6]. 

The purpose of this study is to characterize the long-term, visual outcomes in patients with spasmus nutans and identify comorbid ophthalmic conditions that may impact visual function.

## Methods

This study received approval from the Institutional Review Board of Boston Children’s Hospital, complied with the Health Insurance Portability and Accountability Act, and followed the tenets of the Declaration of Helsinki. Informed consent was waived by the Institutional Review Board.

We undertook a retrospective chart review of patients diagnosed with spasmus nutans at Boston Children’s Hospital who were seen in the Department of Ophthalmology over a 20-year period (2000–2020). In order to identify patients with spasmus nutans, we reviewed the charts of all patients diagnosed with nystagmus by International Classification of Disease ICD-9 and ICD-10 codes over the study period (*n* = 1146). Patients with a diagnosis of spasmus nutans documented in the clinic note on the basis of asymmetric nystagmus with head nodding and/or torticollis were included. Demographic information including gender, self-reported race and ethnicity, type of health insurance, and age at both diagnosis and resolution of spasmus nutans symptoms were recorded. Other relevant historical data were collected.

The baseline examination was defined as the clinic visit at which the patient was diagnosed with spasmus nutans. The final examination was the most recent clinic visit. Examination findings including nystagmus symmetry, head nodding, anomalous head position, ocular alignment, stereopsis, visual acuity, and cycloplegic refraction were recorded from the baseline and final examinations. The visual acuity was converted into LogMAR for analyses. The Preferred Practice Pattern guidelines [7] defined amblyogenic refractive error based on cycloplegic refraction and presence of strabismus. Amblyopia was defined as a ≥ 2 line interocular difference in best-corrected visual acuity or a best-corrected visual acuity of 20/40 or worse in eye(s) that met criteria for an amblyogenic refractive error based on the Preferred Practice Pattern guidelines [7]. Neuroimaging results were collected.

The χ2 test for categorical variables and t-test for continuous variables were used to compare characteristics between follow up groups. Results were deemed statistically significant at *p* < 0.05. All statistical analyses were conducted with Stata/SE, version 15.0.

## Results

We identified 32 children with spasmus nutans among whom 23 had at least two examinations during the study period. The mean follow-up period was 66 months (range 185 months). Mean age at diagnosis of spasmus nutans was 16 months (range 45 months), though the mean age at symptom onset noticed by the caregiver (e.g., head nodding, nystagmus or head turning) was earlier at 8.9 months (range 35 months). Mean age of nystagmus resolution was 48 months (range 114 months). Demographic characteristics are outlined in Table 1. Most patients were male (59%) and not Hispanic or Latino (97%). 66% of patients self-reported as a race other than Caucasian. Nineteen patients (59%) had non-private health insurance or were uninsured (13 Medicaid, 5 accountable care organizations, 1 uninsured). Six patients (19%) had Trisomy 21 and eight patients (25%) had a history of prematurity (gestational age < 37 weeks).

**Table 1:** Demographic characteristics of patients in the study

	Patients (*n* = 32)
Gender	
Male, n (*%*)	19 (59%)
Female, n (*%*)	13 (41%)
Self-Reported Ethnicity	
Not Hispanic or Latino, n (*%*)	31 (97%)
Hispanic or Latino, n (*%*)	1 (3%)
Self-Reported Race	
Caucasian, n (*%*)	11 (34%)
African American or Black, n (*%*)	10 (31%)
Other, n (*%*)	5 (16%)
Thai, n (*%*)	2 (6%)
Asian, n (*%*)	2 (6%)
Dominican, n (*%*)	1 (3%)
Middle Eastern, n (*%*)	1 (3%)
Health Insurance Type	
Private, n (*%*)	13 (41%)
Medicaid, n (*%*)	13 (41%)
Accountable Care Organizations, n (*%*)	5 (16%)
Uninsured, n (*%*)	1 (3%)

Numbers in the table are n (percentage)

The nine patients with spasmus nutans had only one examination despite documented recommendations for follow-up examinations did not differ from those with follow-up examinations with regard to gender, self-reported race and ethnicity, history of prematurity and Trisomy 21, age of diagnosis, and age at resolution of symptoms (*p* values > 0.05). Seven (78%) of the patients lost to follow up had Medicaid insurance, a significantly higher rate compared to the 6/23 patients (26%) with at least two examinations (*p* = 0.007).

### Baseline examination

**Fig. 1:**
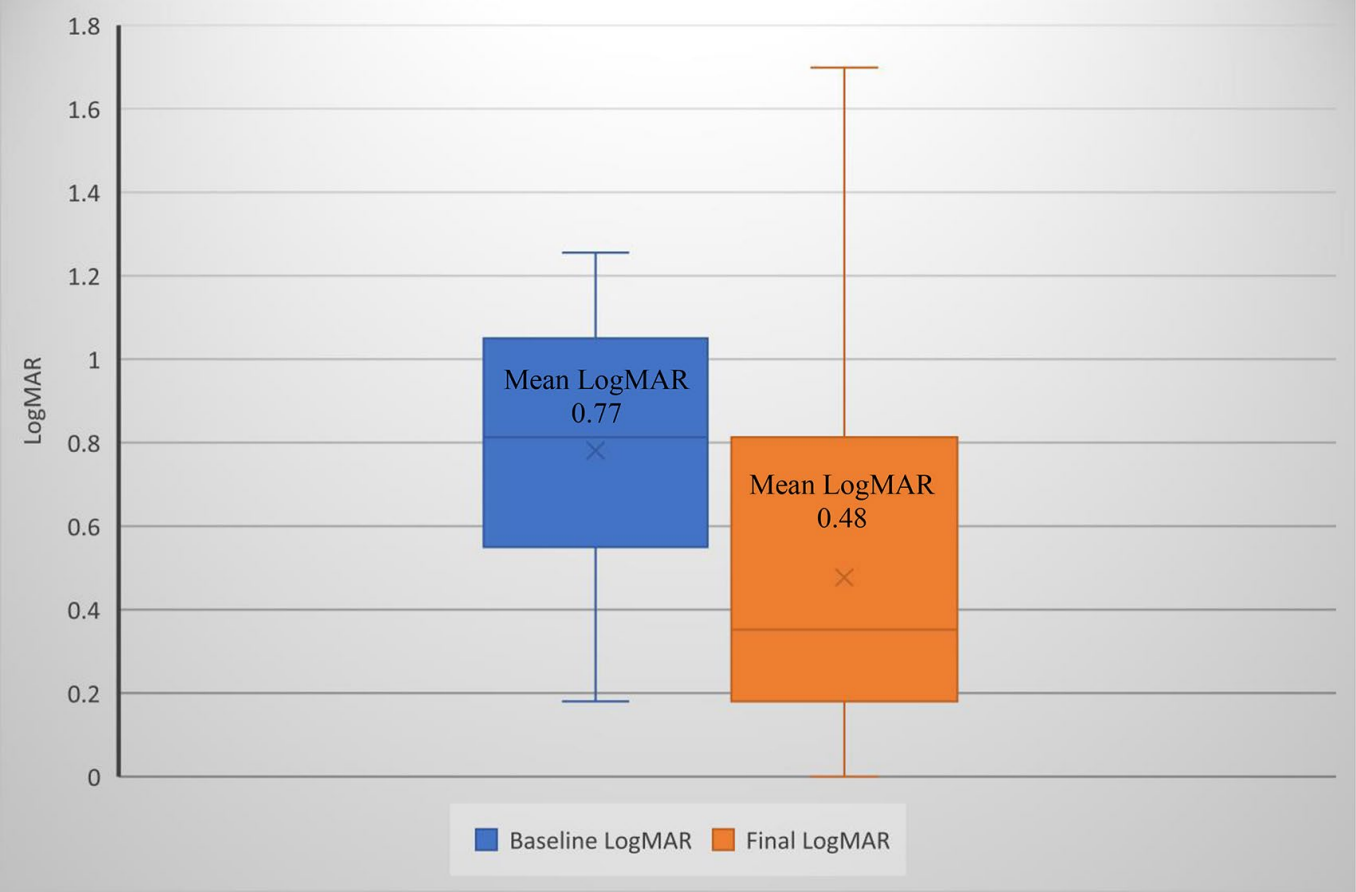
Box and whisker plot of the mean best-corrected visual acuity (LogMAR) in better seeing eye at the baseline and final examination (*n* = 23) in those with > 1 examination. Baseline examination: mean LogMAR 0.77, equivalent to 20/118. Final examination: mean LogMAR 0.48, equivalent to 20/60. The bar displays the interquartile range

All 32 patients had nystagmus (20 characterized as asymmetric), 31 had head nodding, and 16 had ocular torticollis at the baseline examination. Descriptions of the nystagmus and anomalous head position in the study group are provided in Table 2. Six (19%) of the 32 patients had an amblyogenic refractive error; [7] of these, 4 were hyperopic, 1 was myopic, and 1 had amblyogenic astigmatism. Six patients (19%) had strabismus at the baseline exam, all with esotropia; 3 were partially accommodative and 3 were non-accommodative. Two (6%) patients were diagnosed with amblyopia. Of those with > 1 examination, the mean best-corrected visual acuity in the better seeing eye was 0.77 (range 1.29) LogMAR (Snellen equivalent: 20/118) (Fig. 1). Neuroimaging was performed in 23 (72%) patients at a mean age of 18 ± 130 months. No causative, space-occupying lesions were found.

**Table 2:** Description of the nystagmus and anomalous head position at the baseline examination. baseline examination

	Patients (*n* = 32)
Nystagmus	
Asymmetric low to moderate amplitude high frequency horizontal nystagmus, n (%)	6 (19%)
Intermittent very high amplitude low frequency shimmering nystagmus, n (*%*)	5 (16%)
Fine small amplitude high frequency rotary nystagmus, n (*%*)	5 (16%)
Asymmetric very fine nystagmus, n (*%*)	4 (13%)
Asymmetric intermittent fine pendular nystagmus, n (*%*)	4 (13%)
Asymmetric low amplitude low frequency horizontal intermittent nystagmus, n (*%*)	2 (6%)
Asymmetric torsional nystagmus, n (*%*)	2 (6%)
Fine small amplitude pendular nystagmus, n (*%*)	2 (6%)
Asymmetric moderate amplitude pendular nystagmus, n (*%*)	1 (3%)
Asymmetric vertical and rotary nystagmus that dampened at near, n (*%*)	1 (3%)
Anomalous Head Position	
Right head turn, n (*%*)	6 (19%)
Left head turn, n (*%*)	6 (19%)
Right head tilt, n (*%*)	2 (6%)
Chin up position, n (*%*)	1 (3%)
Chin down position, n (*%*)	1 (3%)

Numbers in the table are n (percentage)

Mean LogMAR visual acuity at the baseline examination, decision to perform neuroimaging, and the presence of nystagmus, head nodding, anomalous head position, strabismus, and amblyopia were not significantly different between patients lost to follow up and those with 2 or more examinations (*p* values > 0.05).

### Final examination

A sub-analysis was performed that included patients with greater than one exam (*n* = 23). Final examination ranged from 3 months to 16 years (mean 66 months) after the baseline examination. Eighteen of the 23 patients (78%) had resolved or significantly improved spasmus nutans at the time of the final examination. Of these 18 patients, 3 had nystagmus, 4 had an anomalous head position, and 0 had head nodding. The mean age of the 18 patients who experienced resolved or significantly improved spasmus nutans at the time of the final examination was 7.8 years old with all patients being greater than or equal to 2 years old. Of the 5 patients with persistent spasmus nutans, 3 patients were less than 2 years old and 2 patients were greater than 2 years old (age 3 and 5). Of the five patients with persistent spasmus nutans, 5 had nystagmus, 3 had an anomalous head position, and 3 had head nodding. 12 patients had measurable stereopsis.

**Fig. 2:**
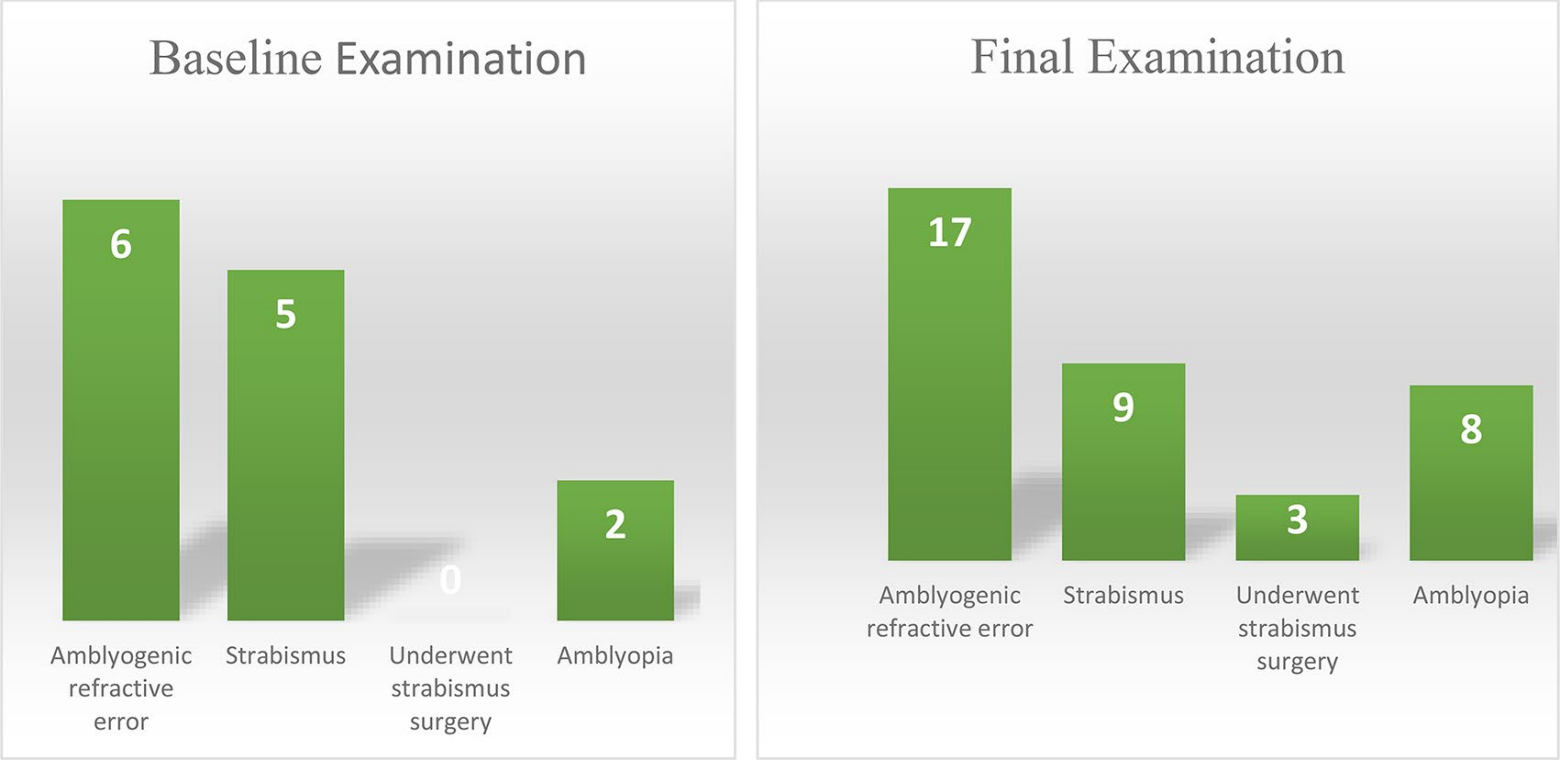
Outcomes at the baseline and final examinations for patients with follow-up data (*n* = 23). Baseline examination: Amblyogenic refractive error: hyperopic (*n* = 4), myopic (*n* = 1), astigmatism (*n* = 1). Partially accommodative esotropia (PAET; *n* = 3); Non-accommodative esotropia (NAET; *n* = 2). Final examination: Amblyogenic refractive error: hyperopic (*n* = 5), myopic (*n* = 6), astigmatism (*n* = 5), anisometropia (*n* = 1). PAET (*n* = 6); NAET (*n* = 3)

Of the patients with a recent cycloplegic refraction at the final exam, 17/20 (85%) had an amblyogenic refractive error (Fig. 2); [7] 6 patients were myopic, 5 were hyperopic, 5 had amblyogenic astigmatism, and 1 had anisometropia.  9 patients (39%) either had strabismus or had a history of strabismus at the final exam, all with esotropia (6 partially accommodative, 3 non-accommodative). 3 (33%) of these patients had undergone strabismus surgery. 8 patients (35%) developed amblyopia. The mean best-corrected visual acuity in the better seeing eye was 0.48 (range 0 to 1.7) LogMAR (Snellen equivalent 20/60) (Fig. 1). 12 patients (52%) demonstrated measurable stereopsis.

Of the 23 patients with > 1 exam, 15 patients at the final follow-up visit were still in the traditional amblyopia treatment period (i.e. less than 8 years old) and 8 patients were beyond the traditional amblyopia treatment period (i.e. 8 years or older). The mean BCVA in the better-seeing eye at the final exam in those less than 8 years old (*n* = 15) was 0.58 LogMAR versus 0.28 in those greater than or equal to 8 years old (*n* = 8).

Of the 6 patients with Trisomy 21, the mean BCVA in the better-seeing eye was  0.40 LogMAR at the final exam (*n* = 6). Of the 8 patients with a history of prematurity, 6 patients had > 1 exam; at this final exam, the mean BCVA in the better-seeing eye was 0.28 LogMAR (*n* = 6).

Trisomy 21 and prematurity are independently associated with strabismus, amblyopia and amblyogenic refractive error. Of the 16 patients without a history of Trisomy 21 and prematurity, 14 patients were able to measure a VA in clinic at the initial exam; the mean BCVA in the better-seeing eye was 0.86 LogMAR at baseline (*n* = 14). Eleven patients without a history of Trisomy 21 and prematurity were seen at the final exam with a mean BCVA in the better-seeing eye of 0.62 LogMAR (*n* = 11). Of these 11 patients, 5 had amblyopia and 3 had strabismus.

## Discussion

Our detailed analysis of ophthalmic outcomes among patients diagnosed with spasmus nutans reveals a high prevalence of amblyogenic refractive error, strabismus, and amblyopia. The prevalence of amblyogenic refractive error [7] increased from 19% to 85% between the baseline and final examinations. Strabismus or history of strabismus was seen in 39% of patients at the final examination, with only half demonstrating measurable stereopsis. Amblyopia was treated in over a third of patients. The high prevalence of these developmental visual disorders among patients diagnosed with spasmus nutans suggests that children may benefit from ophthalmic follow-up until age eight (i.e. past the traditional amblyopic age range) even after manifestations of spasmus nutans have resolved.

Our findings are comparable to similar studies that analyze the characteristics and visual outcomes in spasmus nutans patients. Parikh et al. identified strabismus in 64% of patients (14/22 patients) and amblyopia in 9% of patients (2/22 patients) [6]. Our finding of mean BCVA in the better seeing eye (20/60; range 20/20 to 20/1000 at the final examination is significantly less favorable than that reported by Parikh et al. with their better eyes averaging 20/39 (range 20/20 to 20/200)^6^, and definitely less favorable than Gottlob et al. with their better eyes averaging 20/27 (range 20/20 to 20/50)^3^. Our study found a similar prevalence of Trisomy 21 (19% vs. 18%) as Parikh et al. and a higher prevalence of prematurity (25% vs. 9%)^6^. In an observational case-control study, Wizow et al. found that a lower gestational age was associated with spasmus nutans compared to those with idiopathic infantile nystagmus [5]. 

While the pathogenesis of spasmus nutans is unknown, there are environmental and demographic associations with spasmus nutans that have been identified [5]. These factors include African-American race, Hispanic ethnicity, low gestational age, dark home luminance at birth, parental alcohol and drug abuse, and low socioeconomic status as reflected in lower annual household income, and non-private medical insurance [5]. The majority of patients in our study (66%) self-reported as a race other than Caucasian. Only one patient in our study self-reported as Hispanic. Slightly more than half of patients (59%) had non-private medical insurance or were uninsured. The majority (78%) of patients with only one examination despite a recommendation for a repeat follow-up exam had Medicaid, which was significantly higher than the 26% of patients on Medicaid with at least two examinations. Consequently, spasmus nutans patients with non-private health insurance may be at risk for lost to follow-up.

Our study has several limitations. While our study is the largest of long-term outcomes in spasmus nutans to-date, the rarity of this condition precludes meaningful comparative, subgroup analyses. Further, the retrospective design, necessitated by the rarity of this condition, may serve as a potential source for selection bias and relies on medical documentation with inherent inaccuracies/omissions. Caregivers may not have felt comfortable self-reporting race and ethnicity in particular. The heterogeneous patient population is also a significant limitation, with potential confounding factors that could not be accounted for in the analysis.

## Conclusions

Our study demonstrates a high prevalence of amblyogenic refractive error, strabismus, and amblyopia in patients with a history of spasmus nutans, suggesting that children may benefit from ophthalmic follow-up until they are past the amblyopic age range. Trisomy 21, prematurity, non-Caucasian race, and non-private medical insurance may be associated with spasmus nutans. Patients with spasmus nutans and non-private health insurance may be at risk for becoming lost to follow-up.

### Electronic supplementary material

Below is the link to the electronic supplementary material.

Supplementary Material 1

## Data Availability

All data generated or analyzed during this study is included as a supplementary information file.

## Abbreviations

BCVA: Best-Corrected Visual Acuity
LogMAR: Logarithm of the Minimum Angle of Resolution
ICD: International Classification of Disease
